# The Chemistry of Selenosilanes: A Topic Overview

**DOI:** 10.3390/molecules29194595

**Published:** 2024-09-27

**Authors:** Damiano Tanini, Antonella Capperucci

**Affiliations:** Department of Chemistry “Ugo Schiff”, University of Florence, Via della Lastruccia 3-13, 50019 Sesto Fiorentino, FI, Italy; damiano.tanini@unifi.it

**Keywords:** silyl selenides, heterocycles, nucleophilic substitutions, ring-opening reactions, selenides, diselenides, selenols, metal complexes

## Abstract

Selenium-containing molecules represent a valuable class of compounds with a variety of applications in chemical and biological fields. Selenated reagents are used as intermediates to introduce functional groups (e.g., double bonds) onto different substrates or in the synthesis of various selenated derivatives. Among the variety of selenium-containing reagents, silyl selenides are frequently used to transfer a selenated moiety due to the smooth functionalization of the Se-Si bond, which allows for the generation of selenium nucleophilic species under mild conditions. While the use of the analogous sulfur nucleophiles, namely silyl sulfides, has been widely explored, a relatively limited number of reports on selenosilanes have been provided. This contribution will focus on the application of selenosilanes as nucleophiles in a variety of organic transformations, as well as under radical and redox conditions. The use of silyl selenides to prepare metal complexes and as selenium precursors of materials for atomic layer deposition will also be discussed.

## 1. Introduction

Selenated compounds represent an interesting class of molecules, which are attracting increasing interest for their application in different fields of chemistry [1,2]. They are employed in organic synthesis, in biochemistry (for example, as antioxidants, anticancers and antimicrobials), inorganic chemistry and materials science. Different methods of introducing a selenated moiety into different substrates have been described, and among them, methodologies based on the reactivity of silyl derivatives represent an efficient alternative approach [3,4]. Silyl selenides, in fact, can be regarded as the synthetic equivalents of the corresponding hydrogenated compounds (i.e., RSeSiMe_3_ = RSeH; (Me_3_Si)_2_Se = H_2_Se), but they are more stable and safe and, hence, are easier to prepare, store, handle and measure. In addition, trimethylsilyl selenides, together with trimethylsilyl sulfides, can also be used as soft silylating agents, as well as in the protection of carbonyl groups.

## 2. Synthesis of Selenosilanes

**Scheme 1:**
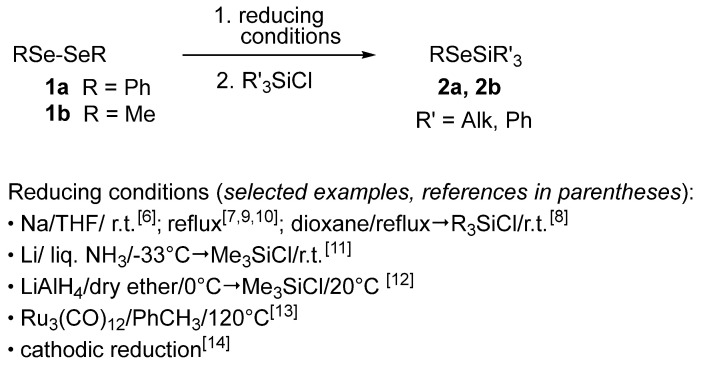
Synthesis of RSeSiR’_3_ **2a**, **2b [6,7,8,9,10,11,12,13,14]**.

Some silyl selenides are commercially available—for example, PhSeSiMe_3_—or can be prepared by different methods [5], mainly depending on the group on the selenium atom. A typical approach for the synthesis of (phenylseleno)trimethylsilane, PhSeSiMe_3_ **2a** or (methylseleno)trimethylsilane, MeSeSiMe_3_ **2b** is the reduction of the parent diselenides **1a**,**b** under suitable reducing conditions (Scheme 1) [6,7,8,9,10,11,12,13,14].

The preparation of butylseleno silanes and oligosilanes was reported by Herzog through the reaction of BuSeLi (obtained from BuLi and Se^0^) with differently substituted chlorosilanes Me_x_Ph_y_SiCl_(4-x-y)_ [10].

**Scheme 2:**
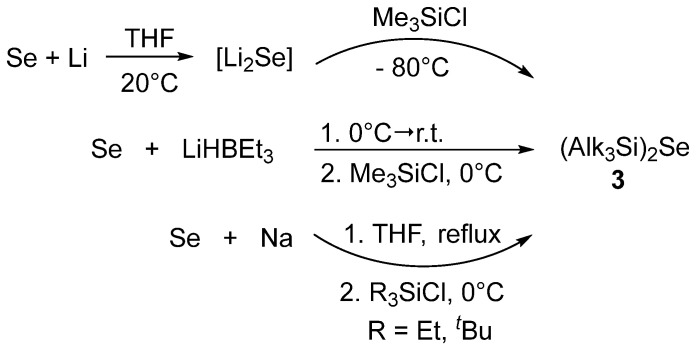
Preparation of bis(trialkylsilyl)selenides **3**.

Bis(trimethylsilyl)selenide (Me_3_Si)_2_Se (hexamethyldisilaselenane, HMDSS) **3a** is synthesized by treating Se(0) with Li(0) [15,16] or with lithium triethylborohydride [17] followed by the addition of the chlorosilane. Silyl selenides with larger groups (Et_3_Si, *^t^*BuMe_2_Si) than Me_3_Si were prepared from Na/Se(0) and the suitable chloroalkylsilane (Scheme 2) [18].

## 3. Selenosilanes in Chemical Synthesis

### 3.1. Silyl Selenides in the Nucleophilic Substitutions on Organic Substrates

#### 3.1.1. Reaction with Halogens or Halogenated Compounds

**Scheme 3:**
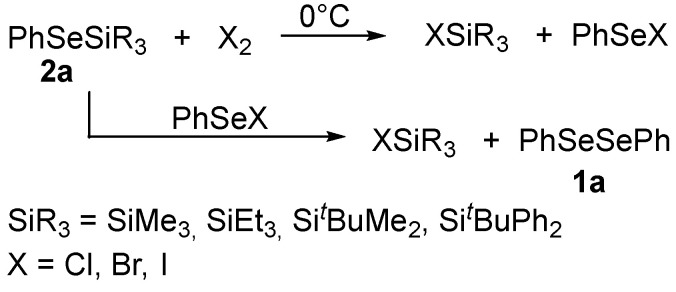
Reaction of **2a** with halogens.

The functionalization of the Si-Se bond under mild conditions enabled the nucleophilic transfer of the seleno moiety onto a variety of organic substrates. In this context, (phenylseleno)trimethylsilane **2a** has been widely used in different organic reactions. Detty and Seidler [19] reported the reaction with halogens to afford silyl halides. The reaction with Cl_2_ was performed in a solvent (CCl_4_ or 1,2-dichlorobenzene), while Br_2_ and I_2_ were used under neat conditions (Scheme 3).

**Scheme 4:**
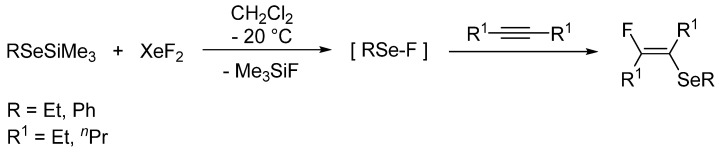
Reaction of xenon difluoride with silylselenides.

Diphenyldiselenide **1a** was formed as a by-product which can be reused to prepare the (phenylseleno)silane. Trialkylsilyl halides were also obtained by the reaction of xenon difluoride with selenosilanes, providing RSe-F intermediates, which were then treated with acetylenes to give selenated fluoro-olefines (Scheme 4) [11].

**Scheme 5:**
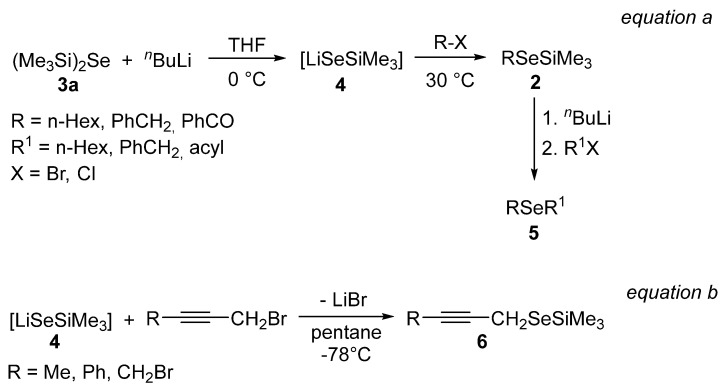
Synthesis of alkyl silyl selenides **5** (*eq. a*) and propargyl selenoethers **6** (*eq. b*).

The treatment of bis(trimethylsilyl)selenide **3a** with *n*-BuLi and alkylation with alkyl halides to provide silyl selenides **2** were reported by Segi and co-workers (Scheme 5, *equation a*) [20]. Further reaction of silyl selenides **2** with *n*-BuLi and alkyl or acyl halides gave unsymmetrical selenides **5** (Scheme 5, *equation a*).

**Scheme 6:**
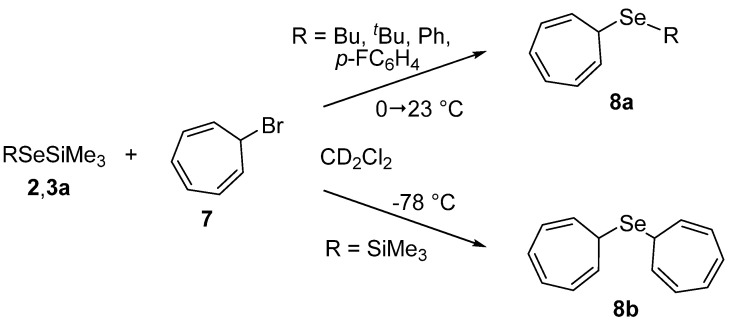
Synthesis of 1-cyclohepta-2,4,6-trienyl-selanes **8**.

Based on a slightly modified procedure, Corrigan described the reaction of [LiSeSiMe_3_] **4** with propargyl bromides to obtain propargyl selenoethers **6** by nucleophilic substitution (Scheme 5, *equation b*) [21]. Characterization by NMR spectroscopy and mass spectrometry were also provided. The reaction of different selenosilanes **2,3a** with tropylium bromide **7** led to 1-cyclohepta-2,4,6-trienyl-selanes **8a,b**, which were characterized by ^1^H, ^13^C and ^77^Se NMR data, including ^1^*J*(^77^Se-^13^C) measurement. The formation of **8b** can be explained through the generation of the tropylium-selenosilane intermediate (C_7_H_7_SeSiMe_3_), which then reacts with **7** to provide selenide **8b**. DFT calculations were also reported (Scheme 6) [22].

#### 3.1.2. Reaction with Benzyl and Allylic Alcohols

**Scheme 7:**
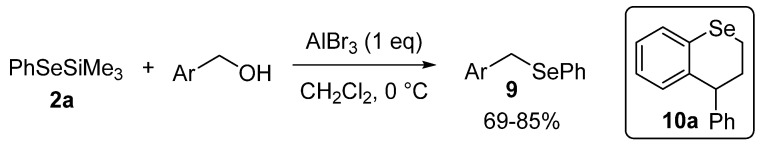
Synthesis of benzyl selenides **9**.

As reported by Abe, Harayama and co-workers, (phenylseleno)trimethylsilane **2a** in combination with a Lewis acid was used as an efficient nucleophile in the direct conversion of benzyl alcohols into benzyl selenides **9** (Scheme 7) [8]. Compared to other Lewis acids (e.g., ZnI_2_, TiCl_4_, AlCl_3_, BF_3_^.^Et_2_O), better results were obtained with AlBr_3_, and higher yields were observed when the PhSeSiMe_3_:AlBr_3_ system was used at a 1:1 ratio.

**Scheme 8:**
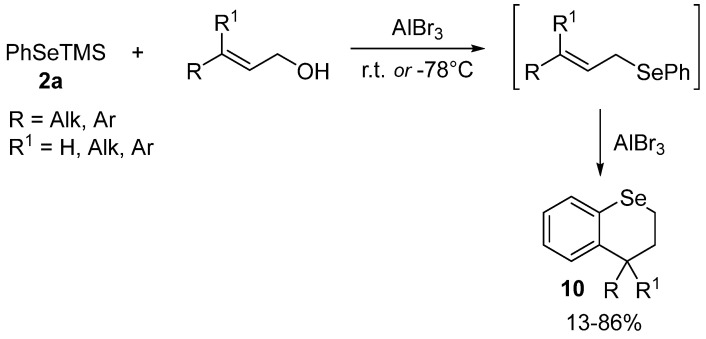
Synthesis of selenochroman derivatives **10**.

When a non-benzylic hydroxy group, such as 2-phenyl ethanol, was reacted, no formation of the expected phenyl(2-phenyl)ethyl selenide was observed, while cinnamyl alcohol afforded the benzoselenane derivative **10a**, formed through a [3,3]-sigma tropic rearrangement of the initially formed selenide PhSeCH_2_CH=CHPh. (Methylseleno)trimethylsilane **2b** behaved as a less efficient nucleophile under the same conditions, leading to the corresponding methyl selenides in rather low yields. The behavior of allylic alcohols was general, as reported by Abe et al. upon reacting differently substituted allylic alcohols with PhSeSiMe_3_/AlBr_3_ to give selenochroman derivatives **10** through a one-pot reaction (Scheme 8) [23].

#### 3.1.3. Reaction with Acetates and Ethers

**Scheme 9:**
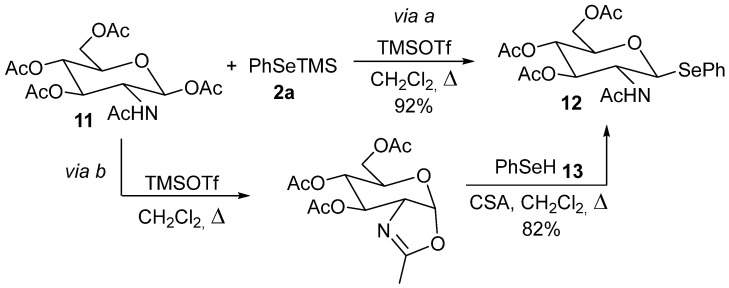
Direct selenoglycosidation of 2-*N*-acetamido-sugars **11** with PhSeSiMe_3_ **2a** (*via a*) or PhSeH **13** (*via b*).

Selenoglycosides represent important intermediates for the synthesis of carbohydrate derivatives. Gallagher and co-workers reported the direct selenoglycosidation of peracetylated amino sugars **11** (galactosamine, mannosamine and glucosamine derivatives) by treatment with (phenylseleno)trimethylsilane **2a** and silyl triflate, providing the corresponding anomeric selenides **12,** as precursors of α-*C*-glycosides, after the substitution of an acetoxy group (Scheme 9, via *a*) [24]. Interestingly, when benzeneselenol **13** was used instead of the selenosilane, in the presence of camphorsulfonic acid (CSA), the formation of the selenoglycosides **12** required a two-step procedure (*via* oxazoline) (Scheme 9, via *b*).

**Scheme 10:**
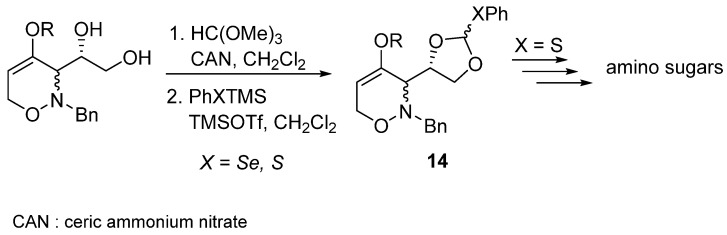
Synthesis of phenylseleno- and phenylthio-substituted 1,2-oxazine derivatives **14**.

In their study on the preparation of amino sugars from 1,2-oxazines, Pfrengle and Reissig reported the reaction of PhSeTMS **2a** (as well as of PhSTMS) to synthesize *syn*- and *anti*-isomers of a 2-phenylseleno (or 2-phenylthio) substituted 1,3-dioxolanes **14** (Scheme 10) [25]. The phenylthio derivative was demonstrated as a precursor of amino sugar derivatives, obtained by a stereodivergent synthesis.

**Scheme 11:**
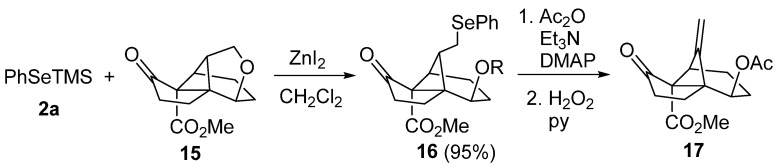
Phenylseleno derivatives as precursor of exocyclic alkenes.

The reaction of a cyclic ether **15** with phenylseleno(trimethylsilane)/ZnCl_2_ was described by Crimmins and Hauser, providing the corresponding selenide **16**, which was converted into the bridged alkene **17** after oxidation and selenoxide elimination (Scheme 11) [26].

#### 3.1.4. Reaction of Selenosilanes with Sulfurated Organic Substrates

**Scheme 12:**
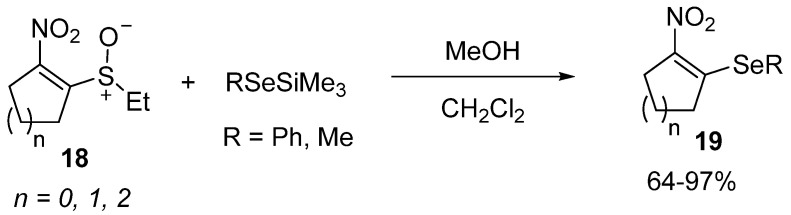
Synthesis of β-selenated nitroalkenes **19**.

β-Heterosubstituted nitroalkenes represent useful synthons in organic chemistry [27]. Taking into account that the sulfinyl group is a good living group, Abe, Harayama and co-workers reported the reaction of β-sulfinyl nitroalkenes **18** with Se-nucleophiles to prepare β-seleno-α,β-unsaturated nitroalkenes **19** [27]. It was found that phenyl selenolate PhSeNa was not able to provide the expected vinyl selenides, while benzeneselenol PhSeH, generated in situ from the corresponding (phenylseleno)trimethylsilane **2a** in methanol, was efficient as a nucleophile. Therefore, the reaction of unsaturated sulfoxides with PhSeSiMe_3_ **2a** and MeOH led to the desired phenyl selenides **19** through a clean addition-elimination reaction (Scheme 12). (Methylseleno)trimethylsilane was also efficient in preparing related methyl selenides in good yields.

**Scheme 13:**
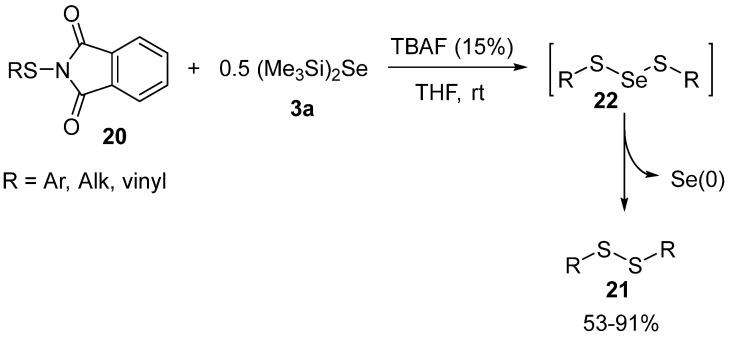
Selenosilane-promoted synthesis of disulfides from *N*-thiophthalimides.

*N*-Thiophthalimides **20** were used as electrophilic sulfur-transfer reagents with bis(trimethylsilyl)selenide **3a** under TBAF catalysis to give variously functionalized disulfides **21** (Scheme 13) [28]. The formation of a selenotrisulfide **22** could be proposed as a possible intermediate, which then provides the substituted disulfides after the elimination of elemental selenium.

#### 3.1.5. Ring Opening of Heterocyclic Rings (O, S, N) by Selenosilanes

**Scheme 14:**
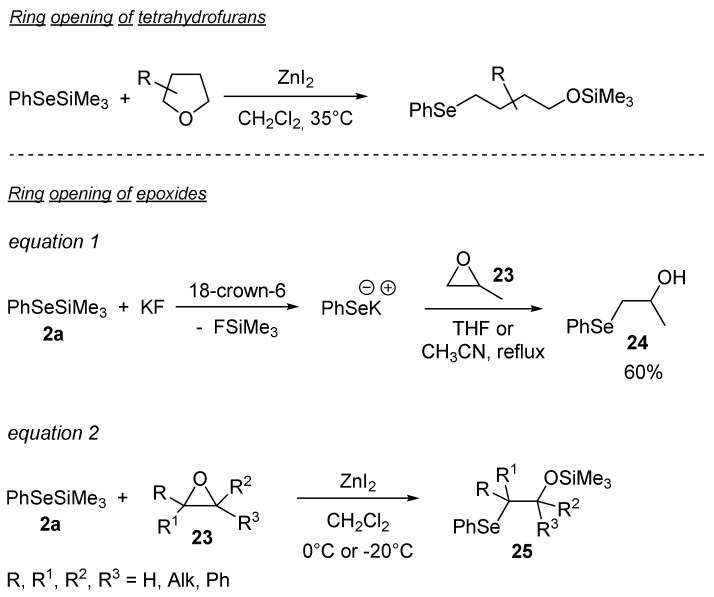
Reaction of PhSeSiMe_3_ with oxygenated heterocycles.

Besides the reaction reported by Crimmins (Scheme 11) [26] and by Murai, Sonoda et al. [29] on the ring opening of tetrahydrofurans by PhSeTMS (Scheme 14 up), most of the examples with oxygenated heterocycles concern the opening of epoxides by selenosilanes. Epoxides **23**, as well as thiiranes and aziridines, are rather reactive species towards nucleophiles for the high strain of the three-membered ring. In this context, β-hydroxy selenides **24** are interesting compounds which find application as versatile intermediates for a variety of synthetic transformations and for their different chemical and biological properties [30]. The nucleophilic substitution of silyl selenides onto epoxides, therefore, represents a convenient method to access this class of bifunctionalized molecules. The ring opening of epoxides **23** by (phenylseleno)trimethylsilane **2a**, as a potassium phenylselenide source, in the presence of KF/18-crown-6, was described by Detty to obtain β-hydroxy selenides **24** (Scheme 14, *equation 1*) [31]. The reaction was also efficient with other organic substrates, such as α,β-unsaturated carbonyls, lactones, esters and halides. Sonoda and Murai reported the reaction under ZnI_2_ catalysis to provide β-siloxyalkyl phenyl selenides **25** (Scheme 14, *equation 2*) [32].

**Scheme 15:**
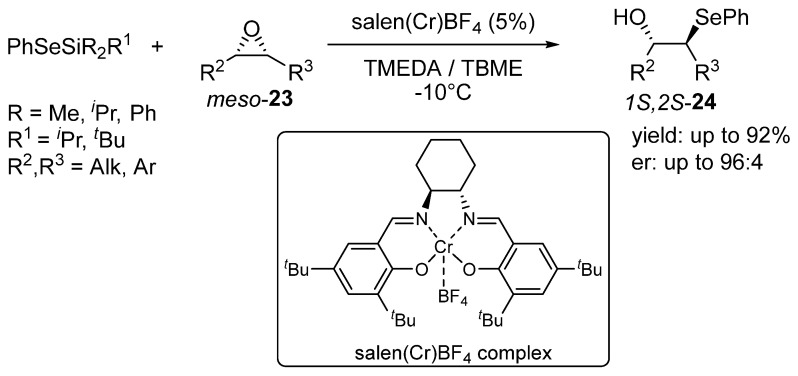
Enantioselective ring opening of *meso*-epoxides by (phenylseleno)silanes.

Enantioselective desymmetrization represents a powerful method to achieve chiral compounds. Organocatalyzed reactions for desymmetrization of epoxides and aziridines with a variety of heteronucleophiles have been quite recently reviewed by Wang [33]. Tiecco and co-workers reported the asymmetric ring opening of *meso*-epoxides (*meso*-**23**) by (phenylseleno)silanes, under salen(Cr)complexes catalysis, to afford various optically active acyclic and cyclic β-hydroxy selenides (*S,S*)-**24** (Scheme 15) [34]. The enantioselectivity of the process depends on the structure of the starting oxirane.

**Scheme 16:**
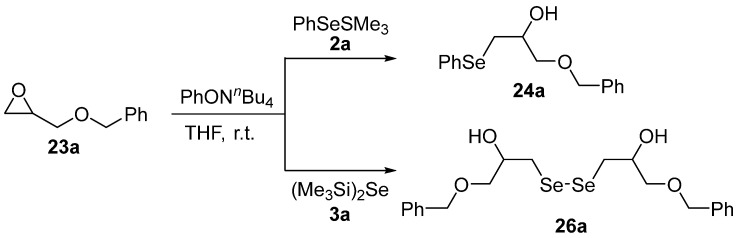
Ring opening of epoxide **23a** by silyl selenides under tetrabutylammonium phenoxide catalysis.

We reported that (phenylseleno)trimethylsilane **2a** efficiently reacted with benzylglycidol **23a** under PhON*^n^*Bu_4_ catalysis, leading to β-phenylselenoalcohol **24a** (Scheme 16), while when bis(trimethylsilyl)selenide **3a** was used, the formation of the corresponding β-hydroxydiselenide **26a** was observed (Scheme 16) [35].

**Scheme 17:**
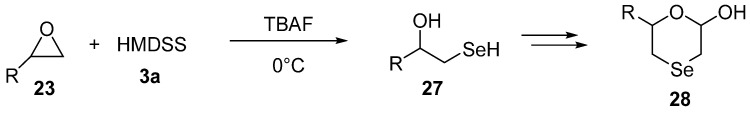
Synthesis of six-membered seleno-heterocycles **28**.

Furthermore, the β-hydroxyselenol **27**, prepared by the reaction of epoxides with HMDSS [36] and treated with a suitable bromo ester, behaved as the precursor of the six-membered chalcogen-containing heterocycles, such as 6-substituted 2-hydroxy 1,4-oxaselenolanes **28**, obtained as a mixture of stereoisomers (Scheme 17) [37].

**Scheme 18:**
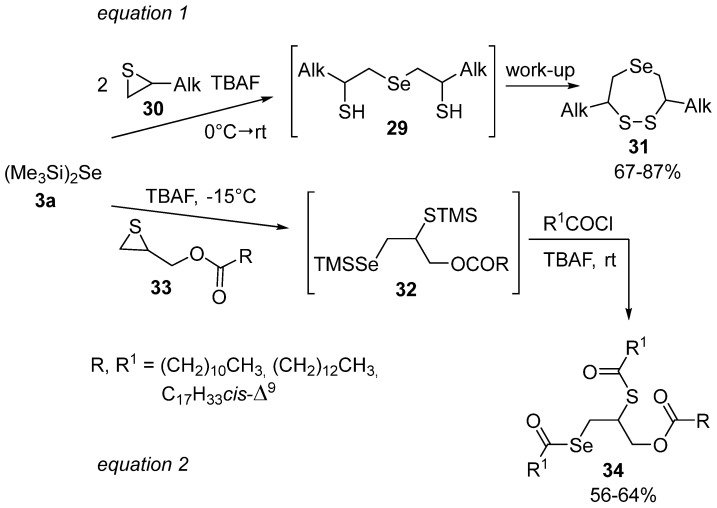
Synthesis of mixed chalcogeno compounds by ring opening of thiiranes **30** and **33** with HMDSS.

The reaction of bis(trimethylsilyl)selenide **3a** (HMDSS) was also performed with thiiranes under TBAF catalysis. Depending on the reaction conditions, from thiiranes **30,** 3,7-disubstituted-1,2,5-dithiaselenepanes **31** were regioselectively obtained (Scheme 18, *equation 1*) [38], reasonably formed by the intramolecular oxidative ring closure of the β-mercapto selenide intermediate **29**. When suitable fatty acid ester-substituted thiiranes **33** of glycidol were reacted with HMDSS/TBAF to afford the bis-silyl intermediate **32**, which was treated with fatty acid acyl chlorides in situ, a regioselective one-pot synthesis of mixed sulfur and selenium isosters of triacyl glycerols **34** was achieved (Scheme 18, *equation 2*) [39]. The physico-chemical properties of these novel fatty acid chalcogeno esters were determined and compared to those of the fully oxygenated triglycerides.

**Scheme 19:**
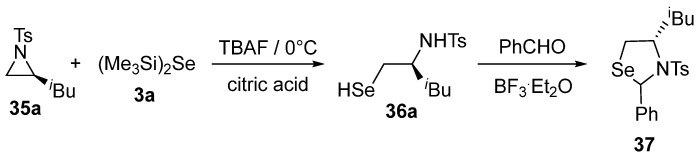
Reaction of bis(trimethylsilyl)selenide with aziridine **35a**.

Our group also found that a *N*-protected aziridine **35a** reacted with HMDSS **3a** to provide a regio- and enantioselective synthesis of the 1,2-amino selenol **36a** (together with the corresponding diselenide) as a precursor of the 2,4-disubstituted 1,3-selenazolidine **37** upon treatment with aldehydes (Scheme 19) [40].

**Scheme 20:**
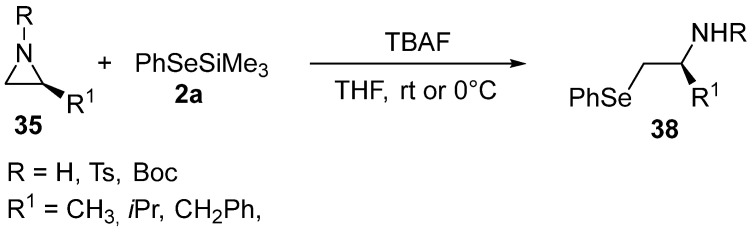
Ring opening of protected and unprotected aziridines by PhSeSiMe_3_.

The regio- and enatioselective ring opening of activated (R = Ts, Boc) and unactivated (R = H) aziridines **35** was also described with (phenylseleno)trimethylsilane **2a** under metal-free conditions, enabling the synthesis of chiral enantioenriched *N*-protected and unprotected β-arylseleno amines **38** (Scheme 20) [41].

**Scheme 21:**
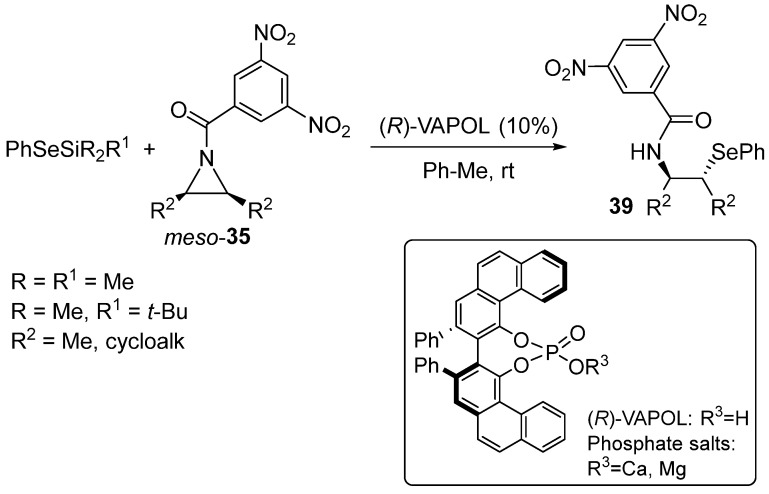
Organocatalyzed desymmetrization of *meso*-aziridines.

In 2011, Della Sala and co-workers reported the first example of the organocatalyzed desymmetrization of *meso*-*N*-acylaziridines (*meso*-**35**) with selenosilanes, promoted by the chiral phosphoric acid (*R*)-VAPOL, leading to β-*N*-acyl-substituted phenyl selenides **39** (Scheme 21) [42].

The silyl selenide with the more sterically hindered silyl group (SiMe_2_*t*-Bu) showed poor reactivity and required a longer reaction time (4–12 days) with respect to the (phenylseleno)trimethylsilane. Better results were obtained when a mixture of PhSeSiMe_3_/PhSeH was used, leading to enantioenriched amine derivatives in a shorter time, high yields and high enantioselectivity. However, it was demonstrated that the silyl-nucleophile was necessary, as the reaction of the same aziridine with (*R*)-VAPOL and the selenol alone gave the product with 45% ee (97% ee with PhSeSiMe_3_/PhSeH). In 2013, the desymmetrization of *meso*-aziridines was re-investigated by Della Sala [43]. It was found that when VAPOL was treated with HCl to have the metal-free chiral phosphoric acid as an organocatalyst, the ring-opening products were formed as racemates in low yields. On the other hand, using a mixture 1:1 of calcium and magnesium phosphate salts of VAPOL, the amine derivatives were obtained in high yields and high ee. Therefore, the metal-free phosphoric acid is not an effective catalyst to promote the desymmetrization of *meso*-aziridines with silyl nucleophiles. The earlier reported results could then be attributed to the action of Ca and Mg phosphate salts, present as unexpected impurities in VAPOL, which could act through a dual Lewis acid–base activation.

**Scheme 22:**
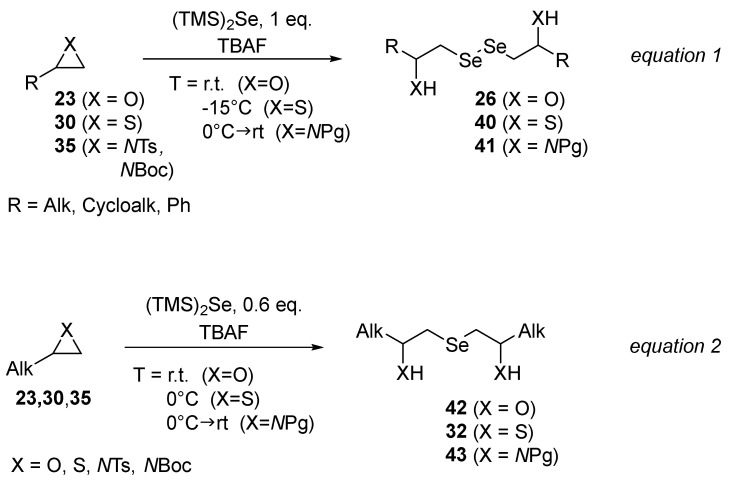
Selenosilane-induced selective synthesis of diselenides and selenides.

As a further step in this study of the behavior of silyl selenides, our group reported the regio- and enantioselective ring opening of epoxides **23**, thiiranes **30** and aziridines **35** by bis(trimethylselenide) **3a**, with TBAF as a catalyst, providing convenient and general access to a variety of β-substituted diselenides **26**, **40–41** (Scheme 22, *equation 1*) and selenides **42**, **32**, **43** (Scheme 22, *equation 2*) through a fine-tuning of the reaction conditions (ratio of reagents, temperature) [44]. The antioxidant catalytic activity of these compounds was also evaluated, with some of them showing a significant glutathione peroxidase (GPx)-like activity. Furthermore, some derivatives proved to be non-toxic, showing no effect on cell viability. The cytotoxicity of selected β-hydroxy selenides was likewise investigated on normal human dermal fibroblasts [45].

In addition, the ring opening of the three-membered heterocycles by (phenylselenotrimethyl)silane was efficiently performed in a variety of ionic liquids, able to act as reaction media and, in some cases, also as catalysts, leading to β-functionalized selenides [46].

**Scheme 23:**
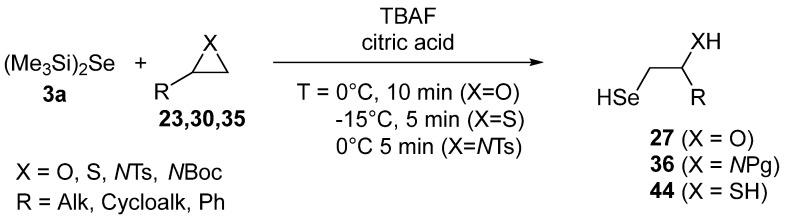
Synthesis of β-substituted selenols.

Extending the scope of this methodology, when strained heterocycles were reacted with bis(trimethylsilyl)sulfide/TBAF under strictly controlled conditions (equivalents of TBAF, time, T), direct access to β-hydroxy, β-mercapto and β-amino alkyl selenols **27**, **36**, **44** was obtained, arising from a regioselective nucleophilic attack on the less hindered side of the heterocycle (Scheme 23) [36]. Interestingly, the ring-opening reaction of enantioenriched substrates provided the synthesis of chiral non-racemic selenols. Taking into account their propensity to be oxidized to diselenides, the β-substituted selenols displayed an unexpected stability, which can be attributed to the hydrogen bond interaction between the selenol and the hydroxy moieties, as indicated by the ab initio DF calculations on selected model systems.

### 3.2. Reaction with C=O Containing Compounds

#### 3.2.1. Reaction with Aldehydes and Ketones

**Scheme 24:**
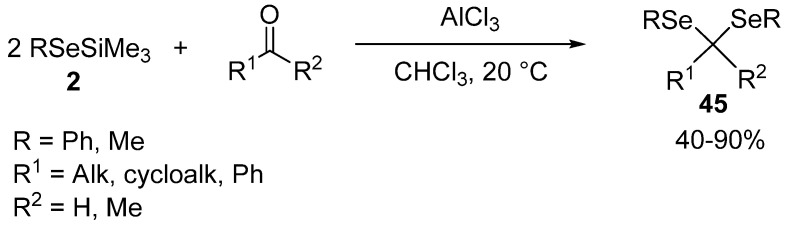
Synthesis of selenoacetals and selenoketals.

Selenosilanes were efficiently reacted with carbonyl compounds to provide selenoacetals **45**, which are used as valuable reagents in organic reactions. Krief et al. reported the selenoacetalization of aldehydes and ketones with selenosilanes under acidic conditions (Scheme 24) [12,47]. Based on the oxygenophilic character of silicon, silylselenides were expected to react without any catalysts. Differently from what was observed with the sulfurated analogs of RSSiMe_3_ (and also with selenoboranes), it was found that the cleavage of the Se-Si bond required an acid catalyst to give the selenoacetalization. Better results were obtained by the in situ formation of RSeSiMe_3_ (prepared by diselenide/LiAlH_4_/ClSiMe_3_) followed by the addition of the carbonyl compound under Lewis acid catalysis.

**Scheme 25:**
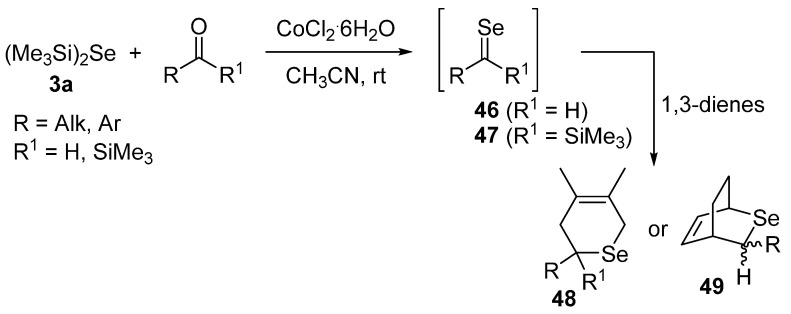
Synthesis of selenoaldehydes and selenoacylsilanes.

The use of silylselenides avoids employing selenols, which are known to be rather unstable. This is important in particular for methylselenol, also because of its high volatility and bad smell. Our group reported the reaction of bis(trimethylsilyl)selenide **3a** with aldehydes and acylsilanes, which in the presence of CoCl_2_^.^6H_2_O afforded selenoaldehydes **46** and selenoacylsilanes **47**, isolated as Diels–Alder cycloadducts **48**, **49** (Scheme 25) [48].

**Scheme 26:**
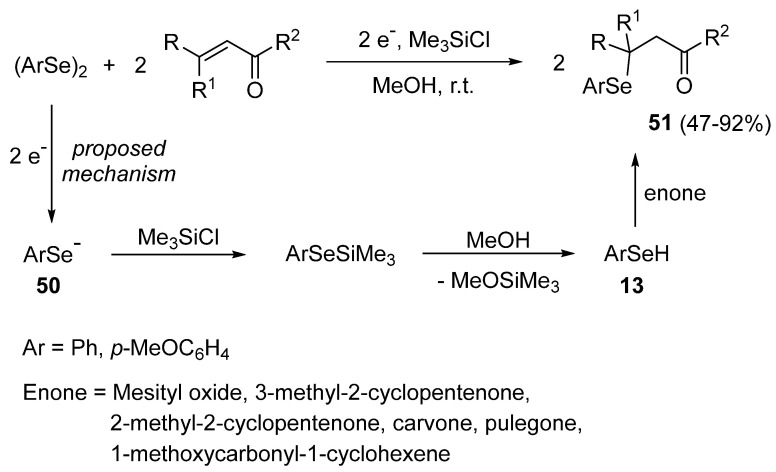
Aryl selenenylation of enones with diselenides/Me_3_SiCl by electroreductive procedure.

Selenenylation of α,β-unsaturated carbonyls by electrolysis with diaryl diselenides and chlorotrimethylsilane was reported by Torii and co-workers [49]. Aryl selenide anions **50** are electrochemically generated using a Pt electrode in a methanolic solution and treated with the enone, the diselenide and Me_3_SiCl (Scheme 26). The reaction affords the β-seleno substituted carbonyl compounds **51** through the mechanism proposed in Scheme 26. Aryl selenols **13** are formed in situ as precursors of the selenated adducts **51**. Furthermore, the addition of the chlorosilane was crucial since without this reagent or by using less than one equivalent, only the starting material was recovered.

**Scheme 27:**
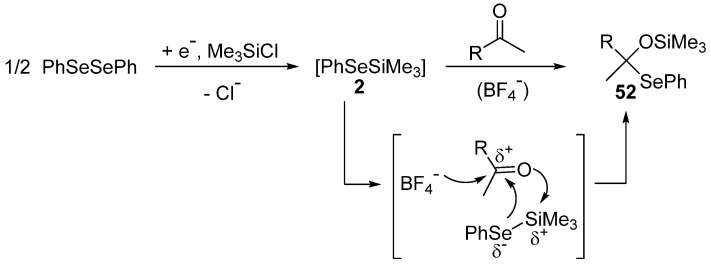
Electrochemical reduction of diphenyldiselenide in the presence of Me_3_SiCl and carbonyls.

A similar reaction was reported by Jouikov et al. when dealing with the cathodic reduction of diselenides (and disulfides) to form PhSe^−^ (or PhS^−^) anions, which in the presence of trimethylchlorosilane gave the corresponding (trimethylsilyl)selenides **2** (and sulfides) in good yields (Scheme 27) [14]. Treatment with carbonyl compounds resulted in the formation of silyl ethers **52** of hemiseleno- (or hemithio-) ketals and acetals. It was also found that the functionalization of carbonyls was preferably performed using silylselenides in a one-pot procedure without their isolation.

When a mixed dichalcogenide PhSSePh was reduced under electrolytic conditions, the rate of the S_N_2 reaction of the PhSe^-^ anion on the Si-Cl bond was faster than the attack of the PhS^−^ anion. This is in agreement with the stronger nucleophilic character of the selenolate anion compared to the thiolate due to the larger size of Se (ionic radius = 198 pm vs. 184 pm for sulfur) and, therefore, the greater localization of the negative charge on Se in the PhSe^−^ species.

**Scheme 28:**
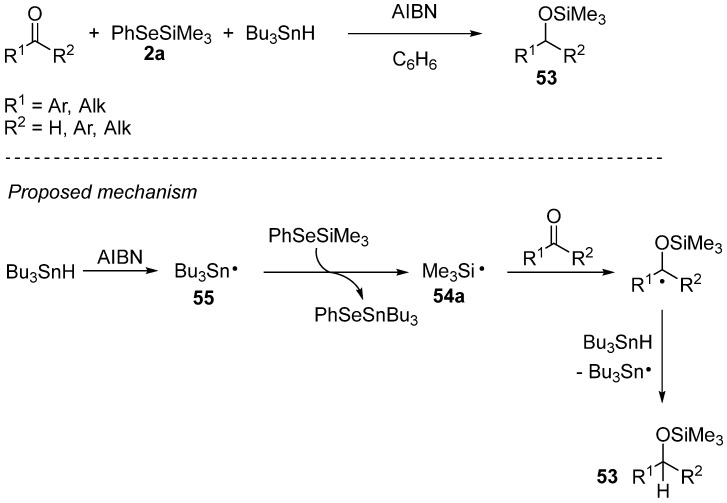
Hydrosilylation of carbonyls with the PhSeSiMe_3_/Bu_3_SnH/AIBN system.

Sonoda and co-workers reported the hydrosilylation of carbonyl compounds under radical conditions to obtain silyl ethers **53**, formed through the treatment of carbonyls with (phenylseleno)trimethylsilane **2a**, tributylstannyl hydride and AIBN (Scheme 28) [50]. On the basis of the proposed mechanism, a silyl radical **54a** is formed in situ by the activation of Se-Si bonds with the stannyl radical **55**.

#### 3.2.2. Reaction with Acyl Chlorides

**Scheme 29:**
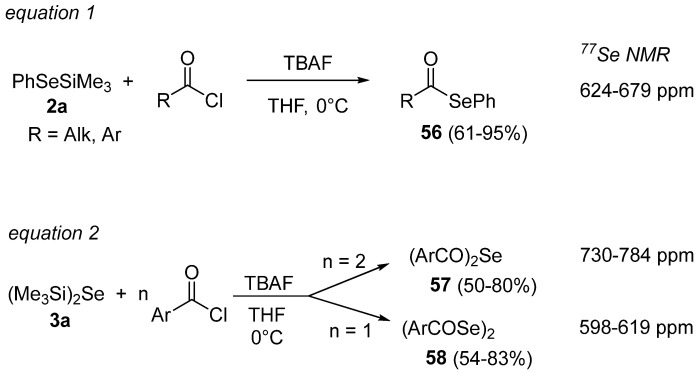
Selective access to selenolesters **56**, selenoanhydrides **57** and diacyl diselenides **58**.

Silyl selenides were also reacted with acyl chlorides, leading to a selective synthesis of selenolesters, selenoanhydrides and diacylselenides depending on the type of the selenosilane used and on the stoichiometric ratio of the reagents. Treatment of acyl chlorides, under TBAF catalysis, with (phenylseleno)trimethylsilane **2a**, led to selenolesters **56** (Scheme 29, *equation 1*), while when bis(trimethylsilyl)selenide **3a** was reacted in a 2:1 or 1:1 ratio, selective access to selenoanhydrides **57** or diacyl diselenides **58**, respectively, was achieved (Scheme 29, *equation 2*) [51]. ^77^Se NMR chemical shifts were also reported, showing typical values for these classes of selenated compounds.

**Scheme 30:**
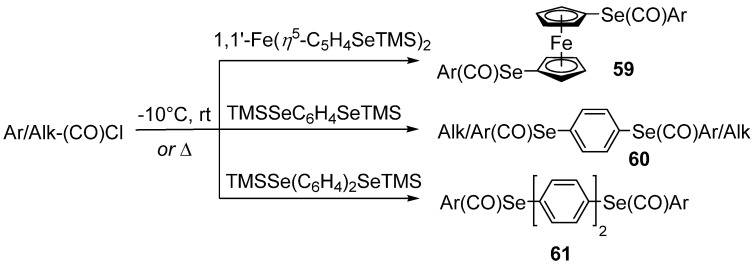
Synthesis of ferrocenyl- and alkyl/arylselenoesters **59−61**.

Besides (phenylseleno)trimethylsilane, Corrigan and Taher reported the reaction of a variety of acyl chlorides with organoselenosilanes containing two TMSSe- groups, such as 1,1’-Fe(*η*^5^-C_5_H_4_SeTMS)_2_, 1,4-TMSSe-C_6_H_4_-SeTMS and 4,4’-TMSSe-(C_6_H_4_)_2_-SeTMS, to afford ferrocenyl- and alkyl/aryl-diselenoesters **59–61** (Scheme 30) [52].

### 3.3. Reduction of Oxides of the Group 16 Elements (S, Se, Te)

**Scheme 31:**
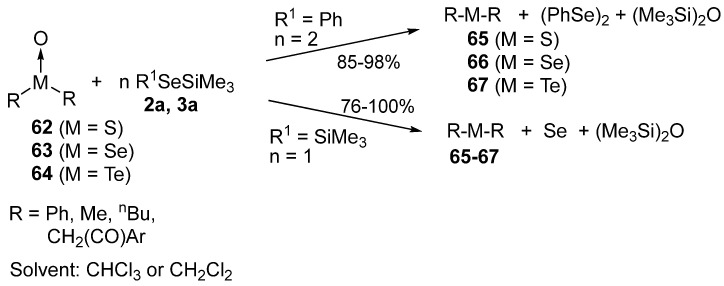
Reduction of S-, Se- and Te-oxides by selenosilanes.

Detty reported the use of (phenylseleno)trimethylsilane **2a** [53] and bis(trimethylsilyl)selenide **3a** [16] to reduce, under mild conditions, sulfoxides **62**, selenoxides **63** and telluroxides **64** to the corresponding sulfides, selenides and tellurides **65–67** in high yields (Scheme 31).

**Scheme 32:**
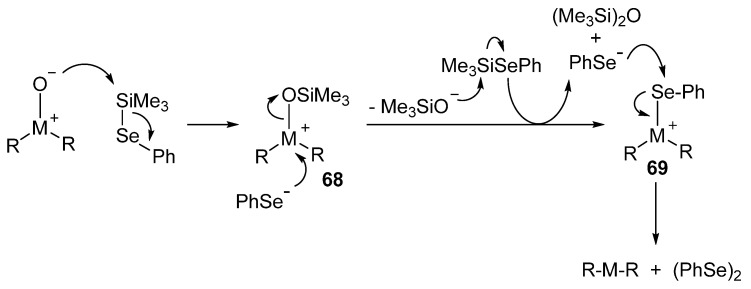
Plausible mechanism for the reduction of chalcogen-oxides.

This method is compatible with different functional groups, such as ketones, phenols, alcohols, olefins, sulfones and nitro derivatives. Based on the proposed mechanism, onium species R_2_M^+^(OSiMe_3_) **68** and R_2_M^+^(SePh) **69** should be involved in this transformation, as depicted in Scheme 32.

### 3.4. Reactivity of Selenosilanes under Radical Conditions

**Scheme 33:**
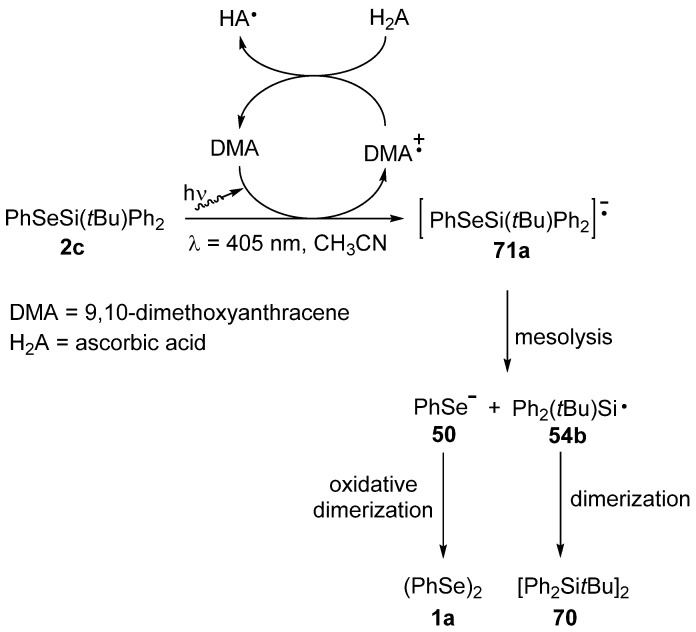
PET activation of **2c** to radical anion **71a** and formation of dimers by mesolysis.

Pandey, Mittal and co-workers investigated the PET (photosensitized electron transfer) promoted activation of selenosilanes to afford radical ions, as well as their fragmentation (mesolysis). *t*-Butyldiphenyl(phenylseleno)silane **2c** was selected for its appreciable stability to study the PET reductive activation of Se-Si bonds using a suitable photosystem to generate the radical anion **71a** (Scheme 33) [54,55].

**Scheme 34:**
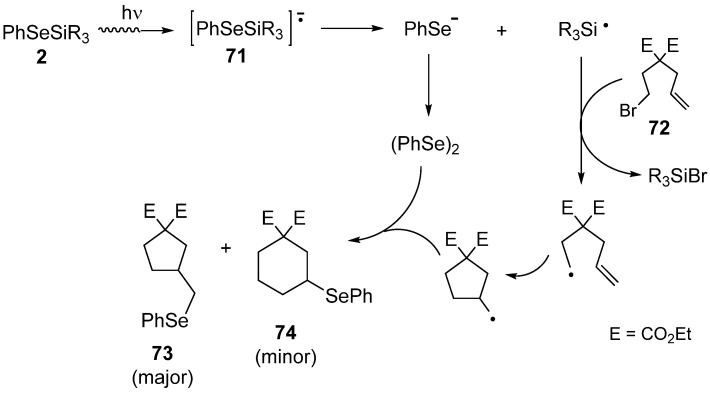
Selenosilanes as BMGT reagents in cyclization reactions.

The formation of the dimers **1a,70** could be rationalized through the mesolysis of the primary radical ion **71a** to form the phenylselenide anion **50** (transient adsorption at λ_max_ = 490 nm) and the silyl radical **54b** (transient adsorption at λ_max_ = 440 nm), which undergo dimerization. It can be assumed that the fragmentation of **71a** is driven by the electronegativity difference between silicon and selenium. This efficient dissociation allowed us to consider selenosilanes as silyl radical equivalents, whose chemical behavior in bimolecular group transfer (BMGT) radical reactions was studied, as well as in intermolecular radical chain transfer addition reactions [54]. For example, for evaluating the use of selenosilanes for BMGT radical reactions, a mixture of compounds **2** and **72** was irradiated together with DMN - 1,5-dimethoxynaphthalene (as an electron donor) and ascorbic acid (as a co-oxidant), which provided the cyclization products **73** (major) and **74** (minor) (Scheme 34).

The reaction was extended to the cyclization of bromoallyl ethers and bromopropargyl ethers and also provided substituted tetrahydrofuran derivatives.

### 3.5. Metal-Selenium Cluster Compounds

**Scheme 35:**
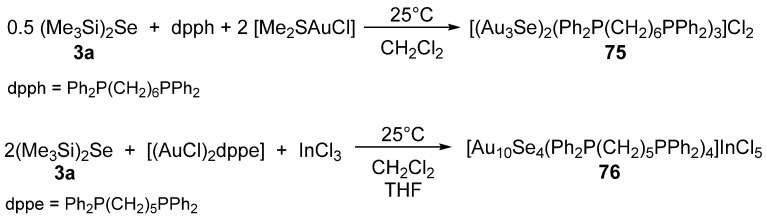
Examples of preparation of some gold–selenium complexes with selenosilanes.

Fenske and co-workers reported the reaction of bis(trimethylsilyl)selenide **3a** with a phosphane ligand (e.g., dpph = Ph_2_P(CH_2_)_6_PPh_2_ or dppe = Ph_2_P(CH_2_)_5_PPh_2_) and [Me_2_SAuCl] to prepare different gold complexes with chalcogenide bridges, as for example in complexes **75** and **76** in Scheme 35 [56]. The structure of some gold–selenium compounds was determined by X-ray diffraction. For instance, complexes **75** and **76** crystallize in the monoclinic space group P21/c and C2/c, respectively, with four molecules per unit cell.

**Scheme 36:**
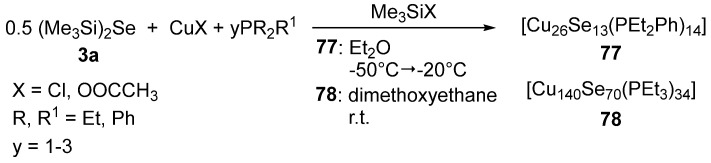
Examples of synthesis of copper selenide cluster molecules.

Fenske also investigated the thermal properties (TGA, DSC) of two series of copper selenide clusters, including structures **77** and **78** depicted in Scheme 36, obtained by the reaction of (Me_3_Si)_2_Se with CuX and PEt_2_Ph or PEt_3_ [57].

**Scheme 37:**
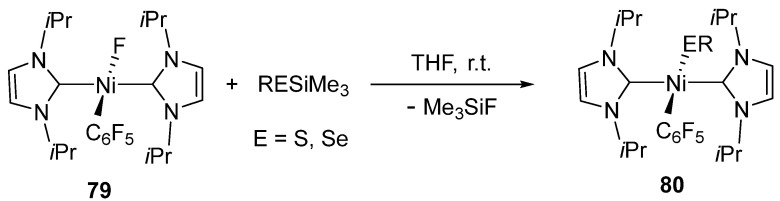
Reaction of complex *trans*-**79** with silyl chalcogenides.

Within a study of the C–F activation in Ni-complexes, Radius et al. investigated the selective replacement of the fluoride ligand by a variety of nucleophiles in the *trans*-[Ni(*^i^*Pr_2_Im)_2_(F)(C_6_F_5_)] complex **79**, which was selected as a model compound. The reaction with silyl chalcogenides (RESiMe_3_, E = S, Se; R = Ph, *^n^*Pr, *^i^*Pr) provided the corresponding sulfurated and selenated complexes **80** with the elimination of fluorotrimethylsilane, favored by the formation of the strong Si-F bond (Scheme 37) [58].

The complexes with *^n^*Pr or *^i^*Pr groups adopt a square planar geometry, as evidenced by single-crystal X-ray analysis. It was found that the Ni-S bond length is slightly shorter than the distance in the related complex [Ni(P*^n^*Bu_3_)_2_(SC_6_F_5_)(C_6_F_5_)] and that the Ni-Se distance was rather unusual.

### 3.6. Silyl Selenides as Se-Precursors for Atomic Layer Deposition (ALD)

**Scheme 38:**
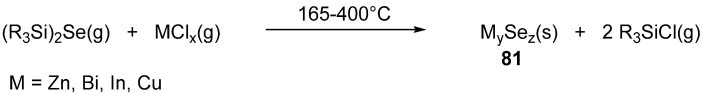
Reaction of silyl selenides with metal chlorides.

In 2009, Pore and co-workers reported the use of bis(trialkylsilyl)selenides (as well as of silyl tellurides) as precursors to obtain metal selenides **81** for atomic layer deposition (ALD) [59], a useful technique to deposit ultra-thin films of a few nanometres in a precise and controlled way for various applications, such as in semiconductors and other nanoscale devices [60]. Silyl selenides are volatile, thermally stable and very reactive towards metal compounds and are thus suitable to produce selenated materials. Compared to alkyl selenides, selenosilanes react more efficiently for the elimination of ligands of the metal precursors. This behavior can be ascribed to the formation of a bond between silicon (hard Lewis acid) and the harder base upon an exchange reaction with metal chlorides. High temperatures are necessary to cause sufficient evaporation of the metal precursors (Scheme 38).

Different combinations of metal precursors and silyl compounds can be used. Besides metal chlorides, growth experiments using Cu(II) pivalate and (Et_3_Si)_2_Se to obtain copper selenides showed that the stoichiometry between CuSe and Cu_2_Se could be controlled, depending on the deposition temperature.

**Scheme 39:**
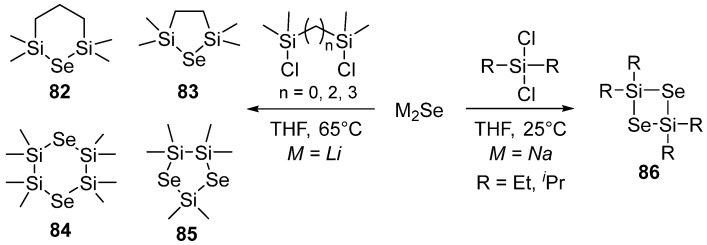
Preparation of cyclic silylselenides.

Bureš and co-workers investigated the behavior of silyl selenides (R_3_Si)_2_Se bearing different alkyl groups on the silicon (R = Me, Et, *^i^*Pr, *^t^*BuMe_2_), evidencing good volatility and stability. The trimethylsilyl substituted silylselenide, in combination with MoCl_5_ as a Mo precursor, was efficiently used for the deposition of MoSe_2_, while the *tert*-butyldimethylsilyl derivative evidenced no atomic layer deposition because of its significant stability [61]. The synthesis of various cyclic silylselenides **82−86**, obtained by in situ treatment of M_2_Se (M = Li, Na) with suitable chlorosilanes, was also reported by Bureš et al. (Scheme 39) [62].

Their thermal behavior was studied by TGA and DSC. TGA showed that they have very good volatility with complete evaporation, while DSC measurements evidenced evaporation without decomposition. The thermal properties mainly depend on the ring size and the number of Si/Se atoms in the ring. The cyclic selenosilanes were evaluated as Se precursors for atomic layer deposition. Combined with MoCl_5_, some of them evidenced a sufficiently fast reaction with metal precursors to permit their application in ALD.

## 4. Conclusions

Selenated compounds represent an interesting class of molecules for their different applications in many fields, such as organic chemistry, inorganic chemistry, materials science, medicinal chemistry and biology. Therefore, methods which allow a mild and general preparation of selenium-containing compounds have received increasing interest. In this regard, selenosilanes have been demonstrated to be efficient reagents that introduce selenated groups on a variety of substrates. The mild functionalization of the Si-Se bond allows silyl selenides to behave as synthetic equivalents of the analogous hydrogenated compounds, but are more stable, less toxic and easier to handle. Thus, differently substituted selenosilanes found a growing number of applications in organic synthesis as versatile nucleophiles towards a variety of organic substrates, being able to undergo chemo-, regio- and stereoselective transformations. Furthermore, selenosilanes are also used in reducing processes and radical reactions, as well as in the preparation of metal clusters and as Se-precursors of metal selenides for atomic layer deposition.

## Data Availability

Not applicable.
